# Transdisciplinary Perspectives on Precision Medicine

**DOI:** 10.1089/heq.2020.0131

**Published:** 2021-05-13

**Authors:** Hector F. Myers, Alecia M. Fair, Fernando Villalta, Katherina Walz, Bettina M. Beech, William K. Scott, David W. Haas

**Affiliations:** ^1^ Department of Medicine, Health & Society, Department of Psychology, and Department of African American & Diaspora Studies, Vanderbilt University, Nashville, Tennessee, USA.; ^2^ Department of Medicine, Division of Geriatric Medicine, Vanderbilt University Medical Center, Meharry-Vanderbilt Alliance, Nashville, Tennessee, USA.; ^3^ Department of Microbiology, Immunology, and Physiology, Meharry Medical College, Nashville, Tennessee, USA.; ^4^ Dr. John T. Macdonald Foundation Department of Human Genetics and John P. Hussman Institute for Human Genomics, Miller School of Medicine, University of Miami, Miami, Florida, USA.; ^5^ Department of Health Systems and Population Health Sciences, University of Houston, College of Medicine, Houston, Texas, USA.

**Keywords:** genomics, social-behavioral, health disparities, knowledge, beliefs

## Abstract

**Purpose:** The Precision Medicine Health Disparities Collaborative fosters collaboration between researchers with diverse backgrounds in precision medicine and health disparities research, to include training at the interface between genomics and health disparities. Understanding how perceptions about precision medicine differ by background may inform activities to better understand such differences.

**Methods:** We conducted a cross-sectional survey of Center members and beyond. Data were collected on categories of educational background, current activities, and level of agreement with 20 statements related to genomics and health disparities. Respondents categorized their background and activities as social/behavioral, genetics, both, or neither. Fisher's exact test was used to assess levels of agreement in response to each statement. Statistically significant associations were further analyzed using ordinal logistic regression adjusting for age, self-identified race/ethnicity, and gender.

**Results:** Of 130 respondents, 50 (38%) identified educational backgrounds and current activities as social-behavioral or genomic 55 (42%). Respondents differed by educational background on the statement Lifestyle and other life experiences influence how genes impact disease risk (*p*=0.0009). Respondents also differed by current activities on the statement Reducing disparities in access to health care will make precision medicine more effective (*p*=0.0008), and on Racism and discrimination make me concerned about how genetic test results will be used (*p*=0.0011).

**Conclusions:** Respondents who differed on prior education and current activities, whether social behavioral science or human genomics, were associated with different perceptions regarding precision medicine and health disparities. These results identify potential barriers and opportunities to strengthen transdisciplinary collaboration.

## Introduction

Progress in health disparities research will benefit from transdisciplinary research that brings together investigators with varied training and professional perspectives. The importance of team science for scientific discovery is well recognized. However, there are challenges with multidisciplinary collaboration, such as different terminologies and methodologies used by different disciplines, which may hinder communication.

Opportunities exist for impact at the interface between precision medicine (including but is not limited to genomics) and social-behavioral research on health disparities. Unfortunately, gaps in training and expertise may cause missed opportunities. Investigators accomplished at genomics may not be knowledgeable in state-of-the-art social-behavioral health disparities research, and vice versa. A better understanding of differing perceptions regarding health disparities among disciplines will improve our ability to leverage precision medicine resources in both genomics and social-behavioral research and, more effectively, help address disparities.

In 2016, the National Institute on Minority Health and Health Disparities (NIMHD) in partnership with the National Human Genome Research Institute (NHGRI) and National Cancer Institute (NCI) established five Transdisciplinary Collaborative Centers (TCCs) for Health Disparities Research Focused on Precision Medicine.^1^ Centers were charged with fostering research emphasizing precision medicine to help address health disparities and fostering collaborations between individuals with expertise in diverse fields, including genomics, medicine, and social-behavioral sciences.^25^ The Centers emphasize translation of discoveries into practice and policy and dissemination of findings to address health disparities.

The Precision Medicine and Health Disparities Collaborative (PMHDC) includes Vanderbilt University Medical Center, University of Miami Medical Center, Meharry Medical College, and University of Mississippi Medical Center. The PMHDC Training and Mentoring Core brings together collaborators from disciplines spanning infectious disease, clinical, health and community psychology, public health, epidemiology, precision medicine, health disparities research, human genomics, and community-based sociocultural research.

Interactions among Center investigators suggested that perspectives regarding precision medicine and health disparities may differ depending on educational background and research experience. We hypothesize that progress in health disparities will be enhanced by focused engagement around concepts and topics where the greatest differences in perspectives exist. To better understand different perspectives relative to educational and professional experience, we developed a survey that asked participants to rate their level of agreement with a series of statements. This survey was designed to gather information regarding perceptions about precision medicine and health disparities research, not to test for knowable facts. This article reports on this pilot project conducted by our Training and Mentoring Core and suggests interpretations of these findings and implications for precision medicine and health disparities research.

## Methods

### Survey development

We conducted a literature review to develop statements that identify differences in belief and perception between those with backgrounds in social-behavioral sciences and genomics. Survey statements were worded to be somewhat provocative to identify areas of disagreement, and to identify opportunities for interdisciplinary discussion.^615^ Item development was based on precision medicine content with a focus on social determinants of health, health disparities and equity, featuring basic facts on genetic research. Survey concepts included, but were not limited, to current level of knowledge and beliefs about precision medicine, knowledge and attitudes about implications of social determinants of health, including racism and discrimination, health care access and utilization, and the utility of genetic testing results in disease risk and treatment response.

### Survey measures

Twenty conceptual statements measuring knowledge, beliefs, perceptions, and attitudes about precision medicine were developed in collaboration between the Center's Consortium and Implementation Cores and the Training and Mentoring Core.^1^ The survey opened with a definition of precision medicine as the use of genetic testing to predict each person's risk of disease, response to treatment, and to guide medical decisions. This may involve testing a huge number of genes in each person. Respondents then self-identified their educational backgrounds and current research activities from 15 disciplines, indicating all that applied, with an option to specify others not listed.

To measure level of agreement with perspectives of precision medicine, 10 statements were developed primarily by Core members with backgrounds in precision medicine and genomics (survey items 1, 2, 3, 6, 8, 14, 15, 16, 18, and 19), while 10 statements were developed primarily by members with backgrounds in social-behavioral science (survey items 4, 5, 7, 9, 10, 11, 12, 13, 17, and 20). Final wording was refined in agreement among all members. Responses to each statement were based on a four-level ordinal scale, with options for *strongly agree*, *agree, disagree,* and *strongly disagree*. A *do not know* response was also allowed. Missing responses were omitted from tests of association of each item with educational background or research activity.

The survey closed with questions about self-identified age, race/ethnicity (American Indian or Alaska Native, Asian, Black or African American, Hispanic or Latino, Native American or Pacific Islander, White, Other, or Mixed), and gender (female, male, and other, with the option to specify other self-identified gender). The survey included an option to provide feedback and follow-up yes/no questions, including, Would you like to give any feedback about this survey? Yes responses prompted the question, May the researchers who designed this survey email you if they have other questions? (see Supplementary Appendix SA1 for survey).

Face validity of survey statements was performed to estimate whether the survey appeared to measure a certain concept. Three coauthors (A.M.F., B.M.B., and K.W.) discussed their independent review of each survey item and then used a dichotomous yes/no scale to distinguish favorable and unfavorable items. Favorable items were objectively structured and could be classified under the predetermined thematic categories. It was determined that the items were good measures to assess general knowledge and subjective impressions of the possible impact of precision medicine.

### Study procedures

We used a single Institutional Review Board (sIRB) for this study as the protocol was standardized across all sites.^1618^ The IRB Reliance Exchange (IREx) was developed by Vanderbilt University Medical Center as the reviewing IRB that provided the sIRB review. Meharry Medical College and University of Miami relied on the review of Vanderbilt's IRB after approval of the initial sIRB application.

To ascertain readability and comprehension of statements, the survey was beta tested with 14 faculty members not affiliated with the PMHDC, from the Vanderbilt Division of Infectious Disease. Information from beta testing feedback was used to finalize the 20 statements.

### Study sample

We conducted a cross-sectional study to survey PMHDC center members at Vanderbilt University Medical Center, Meharry Medical College, and the University of Miami via e-mail using unique links through research electronic data capture.^19^ We felt that it was important to first assess perceptions around precision medicine within our center to better understand the broad scope of definitions of precision medicine.

### Survey deployment

The survey (pilot phase) was deployed to PMHDC center members only (*n*=42) at the above institutions, representing various backgrounds ranging from training in genomics, medicine, public health, clinical, community, and health psychology. Open-ended feedback responses regarding user experience with the survey (feedback and follow-up questions) from our Center members informed several changes to the next iteration of the survey as follows:

(1) The initial definition of Precision Medicine was, Precision Medicine is the use of genetic testing to predict each person's response to treatment, risk of disease, and to guide medical decisions. This may involve testing a huge number of genes in each person. This was changed to, Precision Medicine is an approach for disease treatment and prevention that takes into account variability in each person's genes, environment, and lifestyle. This may involve testing a huge number of genes in each person.
(2) We expanded definitions of human genomics research and social-behavioral science to better capture prior education and current activities: What do we mean by human genomics research?human genomics research may include any laboratory, translational, or clinical research that focuses primarily on human genomics. What do we mean by social-behavioral science? Social and behavioral sciences investigate the interactions between social contexts and processes on behavior and the effects on health.
(3) We expanded self-identified race/ethnicity choices to reflect the Office of Management and Budget Racial and Ethnic Categories and Definitions for National Institutes of Health (NIH) Diversity Programs and for Other Reporting Purposes.
(4) We revised the self-identified gender question to be more inclusive where self-identification is not captured by the female/male dichotomy. The question reads Self-identified gender, with options for male, female, neither, other, or prefer not to answer. An answer of neither or other prompts for further information in free text (see Supplementary Appendix SA1).

The collected survey data and feedback resulted in a second iteration of the survey, which was sent to diverse departments, institutes, and schools (i.e., public health, sociology, psychology, medicine, health and society, genetic medicine, basic sciences, medicine, and dentistry). Eighty-eight individuals (*n*=88) within the PMHDC institutions, but who were not center members, responded.

### Analysis

We followed an *a priori* analysis plan where self-responses to questions about prior training (educational background) and current research activities were used to classify respondents into four categories for educational background and current research: genomics (G), social-behavioral (S/B), both (B), and neither (N). Individuals indicating an educational background or current research in behavioral science or social science, but not genetics, were classified as social-behavioral. Individuals selecting human genomics research, but not behavioral science or social science, were classified as genomics. Those indicating both behavioral science or social science and human genomics research were classified as both. All others were classified as neither. Sample descriptive variables are educational background, current research activities, age, race/ethnicity, and gender.

In the primary analysis, the four groups for educational background and current research activity were compared for differences in response to each of the 20 statements using Fisher's exact test. A secondary analysis was conducted comparing respondents currently engaged in health disparities research with those who were not. We approached each of the 20 statements individually. With 20 statements, Bonferroni-corrected *p*-values <0.0025 were considered statistically significant. Statements with significant association with either educational background or research activity were further analyzed using ordinal logistic regression to adjust for confounding by age, race/ethnicity, and gender and to identify specific groups associated with increasing disagreement with the statement.

## Results

### Description of the sample

A total of 130 unique responses were received from the pilot phase (*n*=42) and second deployment of the survey (*n*=88). For four participants who answered the questionnaire in both the pilot phase and second deployment of the survey, only pilot phase responses were retained. A description of the sample by age, gender, race/ethnicity, educational background, and research area is provided in Table 1. The average age was 51.7 years, the sample was 45% female and 51.6% white, non-Hispanic/Latino. Over half the sample indicated neither social-behavioral nor genetics educational backgrounds or current research areas. About equal proportions reported social-behavioral education (21.5%) or genetics education (20.0%). Similar proportions reported current research in these areas (16.9% social-behavioral, 22.3% genetics). A small proportion reported both backgrounds (4.6%) and current research areas. The distribution of age, gender, and race/ethnicity across educational backgrounds and current research areas did not differ significantly, indicating low likelihood that these factors would confound associations between agreement with the 20 statements and educational background or current research area.

**Table 1.** Demographic and Descriptive Characteristics of Survey Respondents

Characteristic	Overall (*n*=130)
Age in years	
Mean (standard deviation)	51.7 (11.8)
Range	2579
No response	8
Self-identified gender	
Male	70 (54.7%)
Female	58 (45.3%)
No response	2
Self-identified race or ethnicity	
White	66 (51.6%)
Asian	24 (18.8%)
Black or African American	18 (14.1%)
Hispanic or Latino	15 (11.7%)
Other or mixed	4 (3.1%)
American Indian or Alaska Native	1 (0.8%)
No response	2
Categorized self-reported educational background	
Social behavioral	28 (21.5%)
Genetics	26 (20.0%)
Both	6 (4.6%)
Neither	70 (53.8%)
Categorized self-reported research area	
Genetics	29 (22.3%)
Social-behavioral	22 (16.9%)
Both	6 (4.6%)
Neither	73 (56.2%)

Analysis of agreement with the 20 responses and educational background or current research area involved the construction of 44 contingency tables, and significant deviation from random assortment was assessed using Fisher's exact test with simulated *p*-values. The distribution of responses by current research activity is presented in Table 2. Two statements were significantly associated with current research activity. For statement 10, Racism and discrimination make me concerned about how genetic test results will be used, a much larger proportion of individuals engaged in genetics research disagreed or strongly disagreed (44.8%) than any other group (social-behavioral 9.1%, both 16.7%, neither 11.0%). For statement 9, Reducing disparities in access to health care will make precision medicine more effective, the response pattern was complex. No social-behavioral researchers disagreed with the statement, but a larger proportion of individuals engaged in genetics research strongly agreed than overall.

**Table 2.** Survey Responses by Current Research Among Respondents

	Social behavioral (*n*=22)	Human genetics (*n*=29)	Both (*n*=6)	Neither (*n*=73)	Total (*N*=130)	Fisher's exact *p*-value^a^
Q1. Protections are in place that effectively reduce the likelihood of discrimination based on genetic test results.						0.15
Strongly agree	1 (4.5%)	2 (6.9%)	0 (0.0%)	4 (5.5%)	7 (5.4%)	
Agree	7 (31.8%)	20 (69.0%)	2 (33.3%)	26 (35.6%)	55 (42.3%)	
Disagree	4 (18.2%)	4 (13.8%)	3 (50.0%)	23 (31.5%)	34 (26.2%)	
Strongly disagree	1 (4.5%)	0 (0.0%)	0 (0.0%)	7 (9.6%)	8 (6.2%)	
Don't know	9 (40.9%)	3 (10.3%)	1 (16.7%)	13 (17.8%)	26 (20.0%)	
Q2. If a gene variation that predicts drug toxicity in one racial group is present in all racial groups, it will likely predict drug toxicity in all racial groups.						0.84
Strongly agree	0 (0.0%)	1 (3.4%)	0 (0.0%)	3 (4.1%)	4 (3.1%)	
Agree	8 (36.4%)	10 (34.5%)	0 (0.0%)	18 (24.7%)	36 (27.7%)	
Disagree	9 (40.9%)	12 (41.4%)	4 (66.7%)	30 (41.1%)	55 (42.3%)	
Strongly disagree	2 (9.1%)	4 (13.8%)	1 (16.7%)	10 (13.7%)	17 (13.1%)	
Don't know	3 (13.6%)	2 (6.9%)	1 (16.7%)	12 (16.4%)	18 (13.8%)	
Q3. Health benefits of precision medicine outweigh the potential risks to individuals and to populations.						0.13
Strongly agree	3 (13.6%)	10 (34.5%)	0 (0.0%)	15 (20.5%)	28 (21.5%)	
Agree	8 (36.4%)	15 (51.7%)	3 (50.0%)	36 (49.3%)	62 (47.7%)	
Disagree	5 (22.7%)	1 (3.4%)	1 (16.7%)	6 (8.2%)	13 (10.0%)	
Strongly disagree	2 (9.1%)	0 (0.0%)	0 (0.0%)	2 (2.7%)	4 (3.1%)	
Don't know	4 (18.2%)	3 (10.3%)	2 (33.3%)	14 (19.2%)	23 (17.7%)	
Q4. How racial groups are classified is influenced by social, cultural and political factors at least as much as by biological factors.						0.17
Strongly agree	14 (63.6%)	9 (31.0%)	2 (33.3%)	27 (37.0%)	52 (40.0%)	
Agree	6 (27.3%)	14 (48.3%)	4 (66.7%)	39 (53.4%)	63 (48.5%)	
Disagree	1 (4.5%)	5 (17.2%)	0 (0.0%)	4 (5.5%)	10 (7.7%)	
Strongly disagree	1 (4.5%)	0 (0.0%)	0 (0.0%)	2 (2.7%)	3 (2.3%)	
Don't know	0 (0.0%)	1 (3.4%)	0 (0.0%)	1 (1.4%)	2 (1.5%)	
Q5. The degree of genetic diversity is much greater in some racial groups than in other racial groups.						0.047
Strongly agree	6 (27.3%)	15 (51.7%)	1 (16.7%)	16 (21.9%)	38 (29.2%)	
Agree	9 (40.9%)	13 (44.8%)	3 (50.0%)	35 (47.9%)	60 (46.2%)	
Disagree	0 (0.0%)	1 (3.4%)	0 (0.0%)	3 (4.1%)	4 (3.1%)	
Strongly disagree	2 (9.1%)	0 (0.0%)	2 (33.3%)	1 (1.4%)	5 (3.8%)	
Don't know	5 (22.7%)	0 (0.0%)	0 (0.0%)	18 (24.7%)	23 (17.7%)	
Q6. Within the next 5 years I believe that insurance will cover the cost of genetic testing for all diseases.						0.31
Strongly agree	1 (4.5%)	1 (3.4%)	0 (0.0%)	1 (1.4%)	3 (2.3%)	
Agree	8 (36.4%)	7 (24.1%)	1 (16.7%)	18 (24.7%)	34 (26.2%)	
Disagree	7 (31.8%)	13 (44.8%)	3 (50.0%)	29 (39.7%)	52 (40.0%)	
Strongly disagree	2 (9.1%)	6 (20.7%)	2 (33.3%)	7 (9.6%)	17 (13.1%)	
Don't know	4 (18.2%)	2 (6.9%)	0 (0.0%)	18 (24.7%)	24 (18.5%)	
Q7. Socioeconomic differences will affect access to and benefits from precision medicine.						0.17
Strongly agree	18 (81.8%)	16 (55.2%)	6 (100.0%)	40 (54.8%)	80 (61.5%)	
Agree	3 (13.6%)	13 (44.8%)	0 (0.0%)	32 (43.8%)	48 (36.9%)	
Disagree	0 (0.0%)	0 (0.0%)	0 (0.0%)	1 (1.4%)	1 (0.8%)	
Strongly disagree	1 (4.5%)	0 (0.0%)	0 (0.0%)	0 (0.0%)	1 (0.8%)	
Don't know	0 (0.0%)	0 (0.0%)	0 (0.0%)	0 (0.0%)	0 (0.0%)	
Q8. Genetic testing is a reliable way to classify people into meaningful racial groups.						0.20
Strongly agree	0 (0.0%)	1 (3.4%)	0 (0.0%)	3 (4.1%)	4 (3.1%)	
Agree	5 (22.7%)	16 (55.2%)	1 (16.7%)	19 (26.0%)	41 (31.5%)	
Disagree	7 (31.8%)	10 (34.5%)	3 (50.0%)	28 (38.4%)	48 (36.9%)	
Strongly disagree	7 (31.8%)	2 (6.9%)	1 (16.7%)	13 (17.8%)	23 (17.7%)	
Don't know	3 (13.6%)	0 (0.0%)	1 (16.7%)	10 (13.7%)	14 (10.8%)	
Q9. Reducing disparities in access to health care will make precision medicine more effective.						0.0008^*^
Strongly agree	9 (40.9%)	21 (72.4%)	4 (66.7%)	25 (34.2%)	59 (45.4%)	
Agree	11 (50.0%)	7 (24.1%)	0 (0.0%)	39 (53.4%)	57 (43.8%)	
Disagree	0 (0.0%)	1 (3.4%)	2 (33.3%)	2 (2.7%)	5 (3.8%)	
Strongly disagree	0 (0.0%)	0 (0.0%)	0 (0.0%)	0 (0.0%)	0 (0.0%)	
Don't know	2 (9.1%)	0 (0.0%)	0 (0.0%)	7 (9.6%)	9 (6.9%)	
Q10. Racism and discrimination make me concerned about how genetic test results will be used.						0.0011^*^
Strongly agree	12 (54.5%)	3 (10.3%)	2 (33.3%)	23 (31.5%)	40 (30.8%)	
Agree	7 (31.8%)	11 (37.9%)	3 (50.0%)	36 (49.3%)	57 (43.8%)	
Disagree	2 (9.1%)	12 (41.4%)	1 (16.7%)	8 (11.0%)	23 (17.7%)	
Strongly disagree	0 (0.0%)	1 (3.4%)	0 (0.0%)	0 (0.0%)	1 (0.8%)	
Don't know	1 (4.5%)	2 (6.9%)	0 (0.0%)	6 (8.2%)	9 (6.9%)	
Q11. A person's social environment (e.g., poverty, stress burden, and exposure to toxic elements) is a major factor affecting their disease risk.						0.20
Strongly agree	19 (86.4%)	18 (62.1%)	5 (83.3%)	43 (58.9%)	85 (65.4%)	
Agree	3 (13.6%)	10 (34.5%)	1 (16.7%)	28 (38.4%)	42 (32.3%)	
Disagree	0 (0.0%)	1 (3.4%)	0 (0.0%)	1 (1.4%)	2 (1.5%)	
Strongly disagree	0 (0.0%)	0 (0.0%)	0 (0.0%)	0 (0.0%)	0 (0.0%)	
Don't know	0 (0.0%)	0 (0.0%)	0 (0.0%)	1 (1.4%)	1 (0.8%)	
Q12. A person's race is a major contributor to disease risk.						0.69
Strongly agree	2 (9.1%)	2 (6.9%)	1 (16.7%)	8 (11.0%)	13 (10.0%)	
Agree	11 (50.0%)	17 (58.6%)	3 (50.0%)	27 (37.0%)	58 (44.6%)	
Disagree	6 (27.3%)	7 (24.1%)	1 (16.7%)	22 (30.1%)	36 (27.7%)	
Strongly disagree	0 (0.0%)	0 (0.0%)	0 (0.0%)	5 (6.8%)	5 (3.8%)	
Don't know	3 (13.6%)	3 (10.3%)	1 (16.7%)	11 (15.1%)	18 (13.8%)	
Q13. Socioeconomic factors influence access to high quality health care.						0.0077
Strongly agree	22 (100.0%)	23 (79.3%)	5 (83.3%)	46 (63.0%)	96 (73.8%)	
Agree	0 (0.0%)	5 (17.2%)	1 (16.7%)	26 (35.6%)	32 (24.6%)	
Disagree	0 (0.0%)	0 (0.0%)	0 (0.0%)	1 (1.4%)	1 (0.8%)	
Strongly disagree	0 (0.0%)	0 (0.0%)	0 (0.0%)	0 (0.0%)	0 (0.0%)	
Don't know	0 (0.0%)	1 (3.4%)	0 (0.0%)	0 (0.0%)	1 (0.8%)	
Q14. Health benefits that come from precision medicine will be realized equally by all race/ethnicity groups.						0.33
Strongly agree	0 (0.0%)	0 (0.0%)	0 (0.0%)	7 (9.6%)	7 (5.4%)	
Agree	1 (4.5%)	3 (10.3%)	0 (0.0%)	13 (17.8%)	17 (13.1%)	
Disagree	11 (50.0%)	15 (51.7%)	4 (66.7%)	27 (37.0%)	57 (43.8%)	
Strongly disagree	9 (40.9%)	7 (24.1%)	2 (33.3%)	20 (27.4%)	38 (29.2%)	
Don't know	1 (4.5%)	4 (13.8%)	0 (0.0%)	6 (8.2%)	11 (8.5%)	
Q15. A person's genetic makeup is a very important factor influencing their health.						0.079
No response	0	0	0	1	1	
Strongly agree	4 (18.2%)	17 (58.6%)	3 (50.0%)	21 (29.2%)	45 (34.9%)	
Agree	16 (72.7%)	9 (31.0%)	3 (50.0%)	41 (56.9%)	69 (53.5%)	
Disagree	2 (9.1%)	3 (10.3%)	0 (0.0%)	5 (6.9%)	10 (7.8%)	
Strongly disagree	0 (0.0%)	0 (0.0%)	0 (0.0%)	2 (2.8%)	2 (1.6%)	
Don't know	0 (0.0%)	0 (0.0%)	0 (0.0%)	3 (4.2%)	3 (2.3%)	
Q16. Before any genetic test is done on a patient, the patient should be asked to provide written informed consent for the test.						0.056
No response	0	2	0	0	2	
Strongly agree	18 (81.8%)	12 (44.4%)	1 (16.7%)	39 (53.4%)	70 (54.7%)	
Agree	2 (9.1%)	8 (29.6%)	2 (33.3%)	24 (32.9%)	36 (28.1%)	
Disagree	1 (4.5%)	4 (14.8%)	1 (16.7%)	7 (9.6%)	13 (10.2%)	
Strongly disagree	0 (0.0%)	1 (3.7%)	0 (0.0%)	0 (0.0%)	1 (0.8%)	
Don't know	1 (4.5%)	2 (7.4%)	2 (33.3%)	3 (4.1%)	8 (6.2%)	
Q17. A person's education, income, and lifestyle are as important as their genetic makeup in affecting their health.						0.031
Strongly agree	14 (63.6%)	11 (37.9%)	5 (83.3%)	27 (37.0%)	57 (43.8%)	
Agree	7 (31.8%)	14 (48.3%)	0 (0.0%)	36 (49.3%)	57 (43.8%)	
Disagree	1 (4.5%)	0 (0.0%)	1 (16.7%)	7 (9.6%)	9 (6.9%)	
Strongly disagree	0 (0.0%)	0 (0.0%)	0 (0.0%)	0 (0.0%)	0 (0.0%)	
Don't know	0 (0.0%)	4 (13.8%)	0 (0.0%)	3 (4.1%)	7 (5.4%)	
Q18. For most common diseases (e.g., diabetes, hypertension, heart disease), genetic testing is useful in making medical decisions.						0.44
Strongly agree	0 (0.0%)	1 (3.4%)	0 (0.0%)	8 (11.0%)	9 (6.9%)	
Agree	7 (31.8%)	8 (27.6%)	1 (16.7%)	25 (34.2%)	41 (31.5%)	
Disagree	7 (31.8%)	14 (48.3%)	3 (50.0%)	20 (27.4%)	44 (33.8%)	
Strongly disagree	1 (4.5%)	1 (3.4%)	1 (16.7%)	7 (9.6%)	10 (7.7%)	
Don't know	7 (31.8%)	5 (17.2%)	1 (16.7%)	13 (17.8%)	26 (20.0%)	
Q19. Within the next 5 years, genetic testing will greatly improve health outcomes for most chronic diseases (e.g., diabetes, hypertension, heart disease).						0.69
No response	0	0	0	1	1	
Strongly agree	1 (4.5%)	3 (10.3%)	0 (0.0%)	9 (12.5%)	13 (10.1%)	
Agree	5 (22.7%)	7 (24.1%)	1 (16.7%)	22 (30.6%)	35 (27.1%)	
Disagree	10 (45.5%)	11 (37.9%)	2 (33.3%)	20 (27.8%)	43 (33.3%)	
Strongly disagree	2 (9.1%)	3 (10.3%)	1 (16.7%)	5 (6.9%)	11 (8.5%)	
Don't know	4 (18.2%)	5 (17.2%)	2 (33.3%)	16 (22.2%)	27 (20.9%)	
Q20. Lifestyle and other life experiences influence how genes impact disease risk.						0.11
No response	0	0	0	1	1	
Strongly agree	17 (77.3%)	19 (65.5%)	4 (66.7%)	31 (43.1%)	71 (55.0%)	
Agree	4 (18.2%)	10 (34.5%)	2 (33.3%)	32 (44.4%)	48 (37.2%)	
Disagree	0 (0.0%)	0 (0.0%)	0 (0.0%)	3 (4.2%)	3 (2.3%)	
Strongly disagree	0 (0.0%)	0 (0.0%)	0 (0.0%)	0 (0.0%)	0 (0.0%)	
Don't know	1 (4.5%)	0 (0.0%)	0 (0.0%)	6 (8.3%)	7 (5.4%)	

Univariate association of 4-item Likert-type responses (excluding No response and Don't know) with educational background was assessed by Fisher's exact test for a 44 table with a simulated *p*-value obtained from 10,000 permutations.

^a^ Fisher's exact test of 44 table, with No response or Don't know omitted from analysis.

^*^ *p*<0.0025, Bonferroni-corrected threshold for 20 independent tests.

The distribution of responses by educational background is presented in Table 3. Only responses to statement 20, Lifestyle and other life experiences influence how genetics impact disease risk, were significantly associated with educational background. Notably, only individuals with neither social-behavioral nor genetics education disagreed with this statement, and of those that agreed, a smaller proportion strongly agreed.

**Table 3.** Survey Responses by Educational Background Among Respondents

	Social behavioral (*n*=28)	Human genetics (*n*=26)	Both (*n*=6)	Neither (*n*=70)	Total (*N*=130)	Fisher's exact *p*-value^a^
Q1. Protections are in place that effectively reduce the likelihood of discrimination based on genetic test results.						0.096
Strongly agree	1 (3.6%)	1 (3.8%)	0 (0.0%)	5 (7.1%)	7 (5.4%)	
Agree	8 (28.6%)	20 (76.9%)	2 (33.3%)	25 (35.7%)	55 (42.3%)	
Disagree	8 (28.6%)	4 (15.4%)	4 (66.7%)	18 (25.7%)	34 (26.2%)	
Strongly disagree	1 (3.6%)	0 (0.0%)	0 (0.0%)	7 (10.0%)	8 (6.2%)	
Don't know	10 (35.7%)	1 (3.8%)	0 (0.0%)	15 (21.4%)	26 (20.0%)	
Q2. If a gene variation that predicts drug toxicity in one racial group is present in all racial groups, it will likely predict drug toxicity in all racial groups.						0.84
Strongly agree	0 (0.0%)	0 (0.0%)	0 (0.0%)	4 (5.7%)	4 (3.1%)	
Agree	9 (32.1%)	8 (30.8%)	2 (33.3%)	17 (24.3%)	36 (27.7%)	
Disagree	13 (46.4%)	12 (46.2%)	3 (50.0%)	27 (38.6%)	55 (42.3%)	
Strongly disagree	2 (7.1%)	5 (19.2%)	0 (0.0%)	10 (14.3%)	17 (13.1%)	
Don't know	4 (14.3%)	1 (3.8%)	1 (16.7%)	12 (17.1%)	18 (13.8%)	
Q3. Health benefits of precision medicine outweigh the potential risks to individuals and to populations.						0.18
Strongly agree	2 (7.1%)	5 (19.2%)	1 (16.7%)	20 (28.6%)	28 (21.5%)	
Agree	13 (46.4%)	16 (61.5%)	3 (50.0%)	30 (42.9%)	62 (47.7%)	
Disagree	5 (17.9%)	1 (3.8%)	1 (16.7%)	6 (8.6%)	13 (10.0%)	
Strongly disagree	2 (7.1%)	0 (0.0%)	0 (0.0%)	2 (2.9%)	4 (3.1%)	
Don't know	6 (21.4%)	4 (15.4%)	1 (16.7%)	12 (17.1%)	23 (17.7%)	
Q4. How racial groups are classified is influenced by social, cultural, and political factors at least as much as by biological factors.						0.13
Strongly agree	17 (60.7%)	11 (42.3%)	1 (16.7%)	23 (32.9%)	52 (40.0%)	
Agree	9 (32.1%)	10 (38.5%)	5 (83.3%)	39 (55.7%)	63 (48.5%)	
Disagree	1 (3.6%)	4 (15.4%)	0 (0.0%)	5 (7.1%)	10 (7.7%)	
Strongly disagree	1 (3.6%)	0 (0.0%)	0 (0.0%)	2 (2.9%)	3 (2.3%)	
Don't know	0 (0.0%)	1 (3.8%)	0 (0.0%)	1 (1.4%)	2 (1.5%)	
Q5. The degree of genetic diversity is much greater in some racial groups than in other racial groups.						0.34
Strongly agree	8 (28.6%)	13 (50.0%)	1 (16.7%)	16 (22.9%)	38 (29.2%)	
Agree	11 (39.3%)	11 (42.3%)	4 (66.7%)	34 (48.6%)	60 (46.2%)	
Disagree	1 (3.6%)	1 (3.8%)	0 (0.0%)	2 (2.9%)	4 (3.1%)	
Strongly disagree	2 (7.1%)	1 (3.8%)	1 (16.7%)	1 (1.4%)	5 (3.8%)	
Don't know	6 (21.4%)	0 (0.0%)	0 (0.0%)	17 (24.3%)	23 (17.7%)	
Q6. Within the next 5 years I believe that insurance will cover the cost of genetic testing for all diseases.						0.69
Strongly agree	1 (3.6%)	0 (0.0%)	1 (16.7%)	1 (1.4%)	3 (2.3%)	
Agree	10 (35.7%)	5 (19.2%)	1 (16.7%)	18 (25.7%)	34 (26.2%)	
Disagree	9 (32.1%)	14 (53.8%)	2 (33.3%)	27 (38.6%)	52 (40.0%)	
Strongly disagree	3 (10.7%)	5 (19.2%)	2 (33.3%)	7 (10.0%)	17 (13.1%)	
Don't know	5 (17.9%)	2 (7.7%)	0 (0.0%)	17 (24.3%)	24 (18.5%)	
Q7. Socioeconomic differences will affect access to and benefits from precision medicine.						0.018
Strongly agree	22 (78.6%)	16 (61.5%)	6 (100.0%)	36 (51.4%)	80 (61.5%)	
Agree	5 (17.9%)	10 (38.5%)	0 (0.0%)	33 (47.1%)	48 (36.9%)	
Disagree	0 (0.0%)	0 (0.0%)	0 (0.0%)	1 (1.4%)	1 (0.8%)	
Strongly disagree	1 (3.6%)	0 (0.0%)	0 (0.0%)	0 (0.0%)	1 (0.8%)	
Don't know	0 (0.0%)	0 (0.0%)	0 (0.0%)	0 (0.0%)	0 (0.0%)	
Q8. Genetic testing is a reliable way to classify people into meaningful racial groups.						0.12
Strongly agree	0 (0.0%)	0 (0.0%)	0 (0.0%)	4 (5.7%)	4 (3.1%)	
Agree	5 (17.9%)	12 (46.2%)	1 (16.7%)	23 (32.9%)	41 (31.5%)	
Disagree	10 (35.7%)	10 (38.5%)	4 (66.7%)	24 (34.3%)	48 (36.9%)	
Strongly disagree	10 (35.7%)	3 (11.5%)	0 (0.0%)	10 (14.3%)	23 (17.7%)	
Don't know	3 (10.7%)	1 (3.8%)	1 (16.7%)	9 (12.9%)	14 (10.8%)	
Q9. Reducing disparities in access to health care will make precision medicine more effective.						0.012
Strongly agree	13 (46.4%)	16 (61.5%)	4 (66.7%)	26 (37.1%)	59 (45.4%)	
Agree	13 (46.4%)	9 (34.6%)	0 (0.0%)	35 (50.0%)	57 (43.8%)	
Disagree	0 (0.0%)	1 (3.8%)	2 (33.3%)	2 (2.9%)	5 (3.8%)	
Strongly disagree	0 (0.0%)	0 (0.0%)	0 (0.0%)	0 (0.0%)	0 (0.0%)	
Don't know	2 (7.1%)	0 (0.0%)	0 (0.0%)	7 (10.0%)	9 (6.9%)	
Q10. Racism and discrimination make me concerned about how genetic test results will be used.						0.032
Strongly agree	16 (57.1%)	6 (23.1%)	2 (33.3%)	16 (22.9%)	40 (30.8%)	
Agree	9 (32.1%)	10 (38.5%)	2 (33.3%)	36 (51.4%)	57 (43.8%)	
Disagree	2 (7.1%)	8 (30.8%)	2 (33.3%)	11 (15.7%)	23 (17.7%)	
Strongly disagree	0 (0.0%)	0 (0.0%)	0 (0.0%)	1 (1.4%)	1 (0.8%)	
Don't know	1 (3.6%)	2 (7.7%)	0 (0.0%)	6 (8.6%)	9 (6.9%)	
Q11. A person's social environment (e.g., poverty, stress burden, and exposure to toxic elements) is a major factor affecting their disease risk.						0.017
Strongly agree	25 (89.3%)	18 (69.2%)	3 (50.0%)	39 (55.7%)	85 (65.4%)	
Agree	3 (10.7%)	7 (26.9%)	3 (50.0%)	29 (41.4%)	42 (32.3%)	
Disagree	0 (0.0%)	1 (3.8%)	0 (0.0%)	1 (1.4%)	2 (1.5%)	
Strongly disagree	0 (0.0%)	0 (0.0%)	0 (0.0%)	0 (0.0%)	0 (0.0%)	
Don't know	0 (0.0%)	0 (0.0%)	0 (0.0%)	1 (1.4%)	1 (0.8%)	
Q12. A person's race is a major contributor to disease risk.						0.99
Strongly agree	3 (10.7%)	2 (7.7%)	1 (16.7%)	7 (10.0%)	13 (10.0%)	
Agree	13 (46.4%)	13 (50.0%)	3 (50.0%)	29 (41.4%)	58 (44.6%)	
Disagree	7 (25.0%)	8 (30.8%)	2 (33.3%)	19 (27.1%)	36 (27.7%)	
Strongly disagree	1 (3.6%)	0 (0.0%)	0 (0.0%)	4 (5.7%)	5 (3.8%)	
Don't know	4 (14.3%)	3 (11.5%)	0 (0.0%)	11 (15.7%)	18 (13.8%)	
Q13. Socioeconomic factors influence access to high quality health care.						0.0028
Strongly agree	27 (96.4%)	20 (76.9%)	5 (83.3%)	44 (62.9%)	96 (73.8%)	
Agree	1 (3.6%)	5 (19.2%)	1 (16.7%)	25 (35.7%)	32 (24.6%)	
Disagree	0 (0.0%)	0 (0.0%)	0 (0.0%)	1 (1.4%)	1 (0.8%)	
Strongly disagree	0 (0.0%)	0 (0.0%)	0 (0.0%)	0 (0.0%)	0 (0.0%)	
Don't know	0 (0.0%)	1 (3.8%)	0 (0.0%)	0 (0.0%)	1 (0.8%)	
Q14. Health benefits that come from precision medicine will be realized equally by all race/ethnicity groups.						0.19
Strongly agree	0 (0.0%)	0 (0.0%)	0 (0.0%)	7 (10.0%)	7 (5.4%)	
Agree	1 (3.6%)	3 (11.5%)	0 (0.0%)	13 (18.6%)	17 (13.1%)	
Disagree	14 (50.0%)	12 (46.2%)	4 (66.7%)	27 (38.6%)	57 (43.8%)	
Strongly disagree	12 (42.9%)	7 (26.9%)	2 (33.3%)	17 (24.3%)	38 (29.2%)	
Don't know	1 (3.6%)	4 (15.4%)	0 (0.0%)	6 (8.6%)	11 (8.5%)	
Q15. A person's genetic makeup is a very important factor influencing their health.						0.095
No response	0	0	0	1	1	
Strongly agree	6 (21.4%)	16 (61.5%)	3 (50.0%)	20 (29.0%)	45 (34.9%)	
Agree	19 (67.9%)	8 (30.8%)	3 (50.0%)	39 (56.5%)	69 (53.5%)	
Disagree	2 (7.1%)	2 (7.7%)	0 (0.0%)	6 (8.7%)	10 (7.8%)	
Strongly disagree	1 (3.6%)	0 (0.0%)	0 (0.0%)	1 (1.4%)	2 (1.6%)	
Don't know	0 (0.0%)	0 (0.0%)	0 (0.0%)	3 (4.3%)	3 (2.3%)	
Q16. Before any genetic test is done on a patient, the patient should be asked to provide written informed consent for the test.						0.25
No response	0	2	0	0	2	
Strongly agree	21 (75.0%)	11 (45.8%)	3 (50.0%)	35 (50.0%)	70 (54.7%)	
Agree	4 (14.3%)	6 (25.0%)	2 (33.3%)	24 (34.3%)	36 (28.1%)	
Disagree	2 (7.1%)	3 (12.5%)	0 (0.0%)	8 (11.4%)	13 (10.2%)	
Strongly disagree	0 (0.0%)	1 (4.2%)	0 (0.0%)	0 (0.0%)	1 (0.8%)	
Don't know	1 (3.6%)	3 (12.5%)	1 (16.7%)	3 (4.3%)	8 (6.2%)	
Q17. A person's education, income, and lifestyle are as important as their genetic makeup in affecting their health.						0.031
Strongly agree	19 (67.9%)	8 (30.8%)	4 (66.7%)	26 (37.1%)	57 (43.8%)	
Agree	8 (28.6%)	14 (53.8%)	1 (16.7%)	34 (48.6%)	57 (43.8%)	
Disagree	1 (3.6%)	0 (0.0%)	1 (16.7%)	7 (10.0%)	9 (6.9%)	
Strongly disagree	0 (0.0%)	0 (0.0%)	0 (0.0%)	0 (0.0%)	0 (0.0%)	
Don't know	0 (0.0%)	4 (15.4%)	0 (0.0%)	3 (4.3%)	7 (5.4%)	
Q18. For most common diseases (e.g., diabetes, hypertension, heart disease), genetic testing is useful in making medical decisions.						0.42
Strongly agree	0 (0.0%)	0 (0.0%)	0 (0.0%)	9 (12.9%)	9 (6.9%)	
Agree	9 (32.1%)	6 (23.1%)	3 (50.0%)	23 (32.9%)	41 (31.5%)	
Disagree	9 (32.1%)	11 (42.3%)	2 (33.3%)	22 (31.4%)	44 (33.8%)	
Strongly disagree	2 (7.1%)	2 (7.7%)	1 (16.7%)	5 (7.1%)	10 (7.7%)	
Don't know	8 (28.6%)	7 (26.9%)	0 (0.0%)	11 (15.7%)	26 (20.0%)	
Q19. Within the next 5 years, genetic testing will greatly improve health outcomes for most chronic diseases (e.g., diabetes, hypertension, heart disease).						0.83
No response	1	0	0	0	1	
Strongly agree	2 (7.4%)	2 (7.7%)	0 (0.0%)	9 (12.9%)	13 (10.1%)	
Agree	5 (18.5%)	5 (19.2%)	3 (50.0%)	22 (31.4%)	35 (27.1%)	
Disagree	11 (40.7%)	11 (42.3%)	2 (33.3%)	19 (27.1%)	43 (33.3%)	
Strongly disagree	3 (11.1%)	2 (7.7%)	1 (16.7%)	5 (7.1%)	11 (8.5%)	
Don't know	6 (22.2%)	6 (23.1%)	0 (0.0%)	15 (21.4%)	27 (20.9%)	
Q20. Lifestyle and other life experiences influence how genes impact disease risk.						0.0009^*^
No response	1	0	0	0	1	
Strongly agree	21 (77.8%)	19 (73.1%)	5 (83.3%)	26 (37.1%)	71 (55.0%)	
Agree	4 (14.8%)	7 (26.9%)	1 (16.7%)	36 (51.4%)	48 (37.2%)	
Disagree	0 (0.0%)	0 (0.0%)	0 (0.0%)	3 (4.3%)	3 (2.3%)	
Strongly disagree	0 (0.0%)	0 (0.0%)	0 (0.0%)	0 (0.0%)	0 (0.0%)	
Don't know	2 (7.4%)	0 (0.0%)	0 (0.0%)	5 (7.1%)	7 (5.4%)	

Univariate association of 4-item Likert-type responses (excluding No response and Don't know) with educational background was assessed by Fisher's exact test for a 44 table with a simulated *p*-value obtained from 10,000 permutations.

^a^ Fisher's exact test of 44 table, with Don't know responses recoded as missing for analysis.

^*^ *p*<0.0025, Bonferroni-corrected threshold for 20 independent tests.

To examine relationships of particular groups with each of these three statements, ordinal logistic regression models were fit with educational background or current research area as predictor variables (with social-behavioral as the referent category) and age (continuous), gender (female as referent), and race/ethnicity (white as referent) as covariates. Analysis of current research activity statement 10 demonstrated that individuals engaged in genomics research were significantly more likely to disagree with the statement than those engaged in social-behavioral research (*p*=0.000021), controlling for age, gender, and race/ethnicity. For statement 9, no significant differences by current research activity were detected when adjusting for age, gender, and race/ethnicity. For educational background statement 20, individuals with neither social-behavioral nor genetics educational backgrounds were significantly more likely to disagree with the statement than people with social-behavioral education (*p*=0.00024), controlling for age, gender, and race/ethnicity.

In a secondary analysis comparing responses in current health disparities researchers to those not conducting disparities research, none of the 20 statements elicited significantly different patterns of response after correcting for multiple comparisons. The strongest associations were in responses to statement 5, where a larger proportion of current disparities researchers (10.3% vs. 3.9%) disagreed with the statement, The degree of genetic diversity is much greater in some racial groups than in other racial groups, and in responses to statement 9, where only current disparities researchers (9.4% vs. 0%) disagreed with the statement, Reducing disparities in access to health care will make precision medicine more effective.

## Discussion

We presented the results of a survey developed and piloted by our Center to identify areas where perspectives among individuals regarding precision medicine and health disparities research differed depending on categories of educational background and research experience. Perhaps most provocative was the discordant response to the statement, Racism and discrimination make me concerned about how genetic test results will be used. Individuals engaged in genetics research were considerably more likely to disagree with this statement than those engaged in social-behavioral activities, even after controlling for race/ethnicity and other potential confounders.

The overarching motivation for this pilot study was to identify specific survey items to which responses differ substantially in social-behavioral scientists compared to genetics researchers, with categories defined by current research activity and by educational background separately. We did not limit survey distribution to only individuals representing those two groups. In fact, most respondents were neither social-behavioral scientists nor genetics researchers. This afforded us the opportunity to extend analyses to individuals who represented neither of those two groups.

Analyses of the survey data are intended to provide a better understanding of contextual factors that may affect transdisciplinary teams, and help address potential barriers to communication and progress. An ultimate goal is to fill gaps in cross-training between genomic research and health disparities research. This report hopefully provides a somewhat representative view on perceptions regarding precision medicine and health disparities, given that respondents who completed the survey from several institutions represent different races and ethnicities, generally consistent with the percentage of race distribution in the United States,^21,22^ together with their diverse education and expertise in genomics, medicine, public health, social-behavioral sciences, and beyond.

## Conclusions

Several interesting conclusions can be taken from these results. There seems to be more overall agreement than disagreement among participants despite their differing educational background and research activities. Of the 20 survey statements, only 3 identified significant differences between groups, suggesting that most of the statements that were included in our survey may be independent of educational background and research activity, and may be related to other contextual or cultural factors. This may also indicate that a select few statements in this pilot survey were optimal for identifying topics where perspectives differ substantially between groups.

The distribution of responses by educational background showed that answers to the statement, Lifestyle and other life experiences influence how genetics impact disease risk, were significantly associated with educational background, while for the statement Racism and discrimination make me concerned about how genetic test results will be used, engagement in genetics research was significantly associated with disagreement. These findings open a window of opportunity to understand and narrow the knowledge and perception gaps that may delay precision medicine success toward reducing health disparities. Consistent with this need is the answer pattern to the statement, Reducing disparities in access to health care will make precision medicine more effective, where the response pattern was complex with no social behavioral researchers disagreed with the statement, but a larger proportion of individuals engaged in genetics research strongly agreed than overall. The response pattern to this question suggests that the question itself is multifold and should be simplified or more clearly defined for a future study.

This study had several limitations. Most importantly, our analyses are based on a pilot survey with a relatively modest sample size. Expanding this survey to a larger sample size may identify significant associations with additional survey statements within the survey. A larger sample size would also allow us to more effectively control for potential confounders, as well as to perform analyses within subgroups.

### Health equity implications

As we strive for all populations to have equal opportunity to live long, healthy, and productive lives, shaping the current understanding of precision medicine is essential for our Center to help advance progress in addressing health disparities. This stance is heightened as precision medicine has emerged as a promising approach for disease treatment and prevention by accounting for individual variability in genes, environment, and lifestyles. Our aspiration is that precision medicine will help to substantially mitigate or eliminate health disparities, including heart disease, cancer, diabetes, HIV/AIDS, Alzheimer's disease, obesity, COVID-19, and mental illness, that disproportionally affect African Americans and Latinos among other minority groups.^2328^ Results from our survey will positively impact Center members to use new approaches and strategies to advance research and policy decisions in precision medicine by integrating genomics and social-behavioral sciences to address health disparities.

## Supplementary Material

Supplemental data

## Supplementary Material

Supplementary Appendix SA1

## Abbreviations Used

IREx: IRB Reliance Exchange
NCI: National Cancer Institute
NHGRI: National Human Genome Research Institute
NIH: National Institutes of Health
NIMHD: National Institute on Minority Health and Health Disparities
PMHDC: Precision Medicine and Health Disparities Collaborative
sIRB: single Institutional Review Board
TCC: Transdisciplinary Collaborative Center
