# Supplementary material for: Transdisciplinary Perspectives on Precision Medicine
Source: Health Equity. 2021 May 13;5(1):288–98. doi: 10.1089/heq.2020.0131 (PMC8139256; doi:10.1089/heq.2020.0131)
Supplement: Supplemental data [file Supp_Data.pdf]

Perspectives on Precision Medicine (a survey)

Instructions: Please answer each of the following questions to the best of your ability.

You are being asked to participate in this survey research study because your institution is a collaborator with the Center of Excellence in Precision Medicine and Population Health. The purpose of this study is to understand individual's perceptions of precision medicine and use the results of this survey to help identify new training needs in Precision Medicine and Population Health.

1. Your participation in this research study is voluntary. You are also free to withdraw from this study at any time, without penalty or loss of benefits to which you are otherwise entitled. In the event new information becomes available that may affect the risks or benefits associated with this research study or your willingness to participate in it, you will be notified so that you can make an informed decision whether or not to continue your participation in this study.

2. The risks in participating in this study are limited to a potential breach in confidentiality, which is minimized by using this REDCap survey which is a secure HIPAA- compliant data management tool. All efforts, within reason, will be made to keep your personal information in your research record confidential but total confidentiality cannot be guaranteed. Only the PI and study staff will have access to your survey data.

3. The anticipated benefits of this study are furthering our understanding of knowledge and perceptions of precision medicine to better inform educational approaches for Center members regarding precision medicine.

4. Survey participants will not be compensated for participating in this study.

5. If you should have any questions about this research study , please feel free to contact [redacted] For additional information about giving consent or your rights as a participant in this study, to discuss problems, concerns, and questions, or to offer input, please feel free to contact the Institutional Review Board Office at [redacted] or toll free at [redacted]

Please read the following statement:
I have read and understand the above statement, and that by clicking the submit button after completing this survey, I acknowledge that my participation is voluntary.

Description of study procedures: This survey is about your perspectives on precision medicine. You will only receive this survey one time. The survey takes about 5-10 minutes to complete.

Date ______________________________

Signature ______________________________

Name ______________________________

Date of IRB Approval: 01/30/2019

Institutional Review Board

VANDERBILT

E-mail

____

PURPOSE OF THIS SURVEY: To understand how people's perceptions differ about precision medicine. This will help identify new training needs. The survey should take about 5 minutes to complete.

WHAT IS "PRECISION MEDICINE"? Precision Medicine is the use of genetic testing to predict each person's response to treatment, risk of disease, and to guide medical decisions. This may involve testing a huge number of genes in each person.

Which best describe your PRIOR TRAINING (check all that apply)?

- ☐ Behavioral science
- ☐ Clinical research
- ☐ Clinical trials
- ☐ Community engaged research
- ☐ Epidemiology
- ☐ Health disparities
- ☐ Human genomics research
- ☐ Medical care
- ☐ Public health
- ☐ Public policy
- ☐ Social science
- ☐ Translational research
- ☐ Wet lab research
- ☐ Biomedical informatics
- ☐ Law
- ☐ other

Briefly describe your "other" training

____

Which best describe your CURRENT ACTIVITIES (check all that apply)?

- ☐ Behavioral science
- ☐ Clinical research
- ☐ Clinical trials
- ☐ Community engaged research
- ☐ Epidemiology
- ☐ Health disparities
- ☐ Human genomics research
- ☐ Medical care
- ☐ Public health
- ☐ Public policy
- ☐ Social science
- ☐ Translational research
- ☐ Wet lab research
- ☐ Biomedical informatics
- ☐ Law
- ☐ Community member
- ☐ other

Briefly describe your "other" activities

____

Date of IRB Approval: 01/30/2019

Institutional Review Board

VANDERBILT

Please indicate your level of agreement with the following statements

1. Protections are in place that effectively reduce the likelihood of discrimination based on genetic test results.	◯ Strongly agree<br>◯ Agree<br>◯ Disagree<br>◯ Strongly disagree<br>◯ Don't know
2. If a gene variation that predicts drug toxicity in one racial group is present in all racial groups, it will likely predict drug toxicity in all racial groups.	◯ Strongly agree<br>◯ Agree<br>◯ Disagree<br>◯ Strongly disagree<br>◯ Don't know
3. Health benefits of precision medicine outweigh the potential risks to individuals and to populations.	◯ Strongly agree<br>◯ Agree<br>◯ Disagree<br>◯ Strongly disagree<br>◯ Don't know
4. How racial groups are classified is influenced by social, cultural and political factors at least as much as by biological factors.	◯ Strongly agree<br>◯ Agree<br>◯ Disagree<br>◯ Strongly disagree<br>◯ Don't know
5. The degree of genetic diversity is much greater in some racial groups than in other racial groups.	◯ Strongly agree<br>◯ Agree<br>◯ Disagree<br>◯ Strongly disagree<br>◯ Don't know
6. Within the next 5 years I believe that insurance will cover the cost of genetic testing for all diseases.	◯ Strongly agree<br>◯ Agree<br>◯ Disagree<br>◯ Strongly disagree<br>◯ Don't know
7. Socioeconomic differences will affect access to and benefits from precision medicine.	◯ Strongly agree<br>◯ Agree<br>◯ Disagree<br>◯ Strongly disagree<br>◯ Don't know
8. Genetic testing is a reliable way to classify people into meaningful racial groups.	◯ Strongly agree<br>◯ Agree<br>◯ Disagree<br>◯ Strongly disagree<br>◯ Don't know
9. Reducing disparities in access to health care will make precision medicine more effective.	◯ Strongly agree<br>◯ Agree<br>◯ Disagree<br>◯ Strongly disagree<br>◯ Don't know

Date of IRB Approval: 01/30/2019

Institutional Review Board

VANDERBILT

10. Racism and discrimination make me concerned about how genetic test results will be used.	◯ Strongly agree<br>◯ Agree<br>◯ Disagree<br>◯ Strongly disagree<br>◯ Don't know
11. A person's social environment (e.g., poverty, stress burden, and exposure to toxic elements) is a major factor affecting their disease risk.	◯ Strongly agree<br>◯ Agree<br>◯ Disagree<br>◯ Strongly disagree<br>◯ Don't know
12. A person's race is a major contributor to disease risk.	◯ Strongly agree<br>◯ Agree<br>◯ Disagree<br>◯ Strongly disagree<br>◯ Don't know
13. Socioeconomic factors influence access to high quality health care.	◯ Strongly agree<br>◯ Agree<br>◯ Disagree<br>◯ Strongly disagree<br>◯ Don't know
14. Health benefits that come from precision medicine will be realized equally by all race/ethnicity groups.	◯ Strongly agree<br>◯ Agree<br>◯ Disagree<br>◯ Strongly disagree<br>◯ Don't know
15. A person's genetic makeup is a very important factor influencing their health.	◯ Strongly agree<br>◯ Agree<br>◯ Disagree<br>◯ Strongly disagree<br>◯ Don't know
16. Before any genetic test is done on a patient, the patient should be asked to provide written informed consent for the test.	◯ Strongly agree<br>◯ Agree<br>◯ Disagree<br>◯ Strongly disagree<br>◯ Don't know
17. A person's education, income, and lifestyle are as important as their genetic makeup in affecting their health.	◯ Strongly agree<br>◯ Agree<br>◯ Disagree<br>◯ Strongly disagree<br>◯ Don't know
18. For most common diseases (e.g., diabetes, hypertension, heart disease), genetic testing is useful in making medical decisions.	◯ Strongly agree<br>◯ Agree<br>◯ Disagree<br>◯ Strongly disagree<br>◯ Don't know
19. Within the next 5 years, genetic testing will greatly improve health outcomes for most chronic diseases (e.g., diabetes, hypertension, heart disease).	◯ Strongly agree<br>◯ Agree<br>◯ Disagree<br>◯ Strongly disagree<br>◯ Don't know

Date of IRB Approval: 01/30/2019

Institutional Review Board

VANDERBILT

20. Lifestyle and other life experiences influence how genes impact disease risk.	◯ Strongly agree<br>◯ Agree<br>◯ Disagree<br>◯ Strongly disagree<br>◯ Don't know

Your age, race/ethnicity, and gender

Age in years	______________________
Self-identified race/ethnicity	◯ American Indian or Alaska Native<br>◯ Asian<br>◯ Black or African American<br>◯ Hispanic or Latino<br>◯ Native Hawaiian or Other Pacific Islander<br>◯ White<br>◯ other or mixed
Please explain "other or mixed" race/ethnicity	______________________
Self-identified gender	◯ Female<br>◯ Male<br>◯ other
Please explain "other" gender	______________________

Feedback and follow-up

Would you like to give any feedback about this survey?	◯ Yes<br>◯ No
Your feedback	______________________
May the researchers who designed this survey email you if they have other questions?	◯ Yes<br>◯ No

Date of IRB Approval: 01/30/2019

Institutional Review Board

VANDERBILT
